# Non-Targeted Screening of Metabolites in Aqueous-Ethanol Extract from *Spiraea hypericifolia* (Rosaceae) Using LC-HRMS

**DOI:** 10.3390/ijms241813872

**Published:** 2023-09-08

**Authors:** Vera A. Kostikova, Natalia V. Petrova, Tatiana M. Shaldaeva, Vladimir V. Koval, Alexander A. Chernonosov

**Affiliations:** 1 Central Siberian Botanical Garden, Siberian Branch, Russian Academy of Sciences (SB RAS), Novosibirsk 630090, Russia; tshaldaeva@yandex.ru; 2 Komarov Botanical Institute, Russian Academy of Sciences, St. Petersburg 197022, Russia; npetrova@binran.ru; 3 Institute of Chemical Biology and Fundamental Medicine, Siberian Branch, Russian Academy of Sciences (SB RAS), Novosibirsk 630090, Russia; koval@niboch.nsc.ru (V.V.K.); alexander.chernonosov@niboch.nsc.ru (A.A.C.)

**Keywords:** *Spiraea hypericifolia*, LC-HRMS, HPLC, metabolome, flavonoid, antioxidant activity

## Abstract

By means of liquid chromatography combined with high-resolution mass spectrometry, metabolite profiling was performed on an aqueous-ethanol extract from *Spiraea hypericifolia* (Rosaceae) collected in Siberia (Russia). Up to 140 compounds were found in the extract, of which 47 were tentatively identified. The identified compounds were amino acids, sugars, phenylpropanoids, fatty acids and their derivatives, triterpenoids, flavonoids, and others. A quantitative analysis showed the predominance of phenolcarboxylic acids and flavonoids in the studied extract, but a qualitative analysis revealed the higher structural diversity of flavonoids. Of the 23 identified flavonoids, 13 were flavonols: quercetin, hyperoside, isoquercitrin, reynoutrin, avicularin, rutin, quercetin-3-O-(6″-O-malonyl)-β-D-glucoside, 3-O-methylquercetin-3′-O-β-D-glucopyranoside, isorhamnetin, rhamnetin-3-O-β-D-xylopyranosyl-β-D-glucopyranoside, kaempferol, tiliroside, and trifolin; six were catechins: catechin, (−)-epicatechin, (+)-epicatechin, (+)-catechin-7-O-β-D-xyloside, (2S,3R)-3,5-dihydroxy-2-(4-hydroxyphenyl)-3,4-dihydro-2H-chromen-7-yl-β-D-glucopyranoside, and catechin 7-O-apiofuranoside; two are isoflavones: genistin and genistein; and one was a flavone (luteolin-4′-O-β-D-glucopyranoside) and another was an anthocyanidin (pelargonidin). The aqueous-ethanol extract from *S. hypericifolia* showed antioxidant activity (half-maximal inhibitory concentration 102.95 μg/mL), which was likely related to the high concentrations of phenolcarboxylic acids (229.6 mg/g), flavonoids (118.3 mg/g), and tannins (62.9 mg/g).

## 1. Introduction

**Figure 1:**
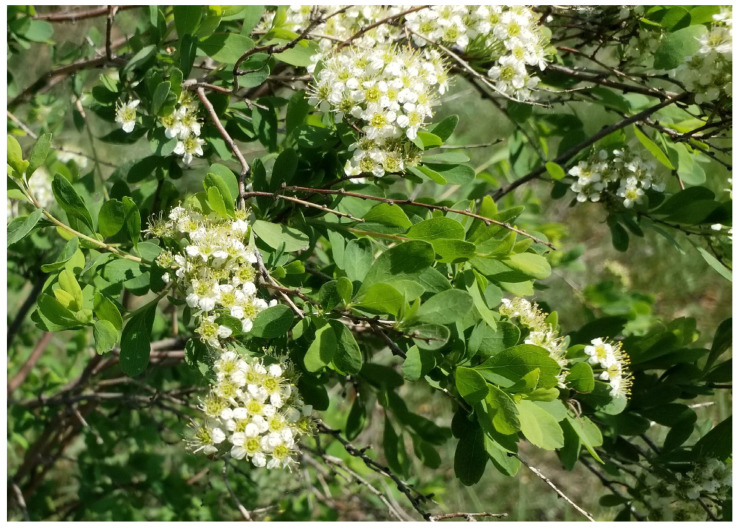
*Spiraea hypericifolia*; photo by Vera A. Kostikova.

*Spiraea hypericifolia* L. is affiliated with the family Rosaceae Juss., the section *Chamaedryon* Ser. in the *Spiraea* L. genus, which, according to various estimates, includes 80 to 120 taxa. This shrub is up to 150 cm high and has long, brown, glabrous, rod-like branches (Figure 1). The young shoots are glabrous or pubescent, cylindrical, and ribbed. The leaves are entire, ranging from pointed oblongly ovate and lanceolate to narrowly obovate; they are wedge-shaped narrowed at the base, smooth-margined, on sterile shoots that usually have several denticles at the apex, and grayish. The flowers are white, and simple sessile umbrellas contain 4–16 of them each. The fruit is a follicle. It grows as stand-alone specimens or in small groups on steppe rocky slopes of mountains, in flat steppes, on cliffs, and on stony placers. If there is a shortage of moisture in desert–steppe areas, then this plant forms dwarf bushes (10–20 cm high) with tiny leaves. It can be found in the Sayan Mountains up to 1400 m above sea level and in the Altai and Tuva up to 1650 m above sea level [1,2]. The bulk of *S. hypericifolia*’s geographical range is situated in Eurasia, and this plant is considered one of the most evolutionarily developed members of this genus [3]. Species of the genus *Spiraea*, including *S. hypericifolia*, are ornamental plants and are often used to decorate gardens and parks [1].

Species of the genus *Spiraea* are known as valuable ornamental plants, and for some of these species, the biological activity of their extracts and individual compounds has been documented, including anti-inflammatory, antioxidant, antiviral, antibacterial, antifungal, anticancer, and other effects [4,5,6,7,8,9,10]. *Spiraea hypericifolia* is one of these species. In Tibetan medicine, the bark, roots, and leaves of *S. hypericifolia* are employed to treat rheumatoid arthritis, gastrointestinal diseases, gynecological diseases, and helminthiases, while in traditional Kazakh medicine, they are used for treating dermatoses. The leaves show antibacterial activity. An infusion of flowers is employed to treat algomenorrhea [7]. Flavans extracted from *S. hypericifolia* possess relatively weak toxicity and exert anticancer actions both on their own and in combination with radiotherapy in in vitro and in vivo studies. The fungicidal effects of the phenolic acids found in *S. hypericifolia* leaves have been documented [8]. Apparently, the combination of various biologically active compounds in extracts from *S. hypericifolia* gives useful pharmacological properties to this species. Previously, we studied the antioxidant and anticancer potential of this plant from other populations [6,9,10]. It was also reported elsewhere that a dry extract (DE) from the aboveground part of *S. hypericifolia* studied in the current work has the best anti-influenza effects among investigated *Spiraea* species in the Asian part of Russia: the virus neutralization index (a logarithmic metric) is 4.0 toward the human influenza virus and 4.25 toward avian influenza virus, and this plant is one of the least toxic *Spiraea* species (the highest concentration tolerable for MDCK cells in culture is 0.5 mg/mL) [9]. The antioxidant effect and the full profile and levels of metabolites in this DE have not been determined before. Metabolic analysis has become one of the tools for solving ecological, taxonomic, biochemical, physiological, and other problems [11]. It is used in those scientific fields where a search for patterns and interdependencies is problematic. The approaches involving metabolomics are based on an analysis of the qualitative and quantitative profiles of all extractable compounds as well as their correlations. This method is suitable for the purposes of pharmacology, where plant extracts as a whole are often used rather than individual compounds. Metabolomic analysis has not been previously performed on *Spiraea* species (in particular, on *S. hypericifolia*).

The aim of this study was to quantitatively analyze the aqueous-ethanol DE from *S. hypericifolia* for biologically active metabolites by liquid chromatography coupled with high-resolution mass spectrometry (LC-HRMS) and by high-performance liquid chromatography (HPLC), as well as to evaluate its antioxidant activity.

## 2. Results and Discussion

### 2.1. LC-HRMS Analysis of Bioactive Compounds in the S. hypericifolia Extract

By LC-HRMS, chromatographic profiles of the aqueous-ethanol extract of the aerial part of *S. hypericifolia* were obtained and investigated. This analysis allowed us to separate up to 140 compounds in the extract, which were tentatively identified by exact mass using several databases of compounds. The 47 most reliably identified compounds that matched a fragmentation pattern in the mzCloud database or standards are presented in Table 1.

The analysis of metabolites in the retention time interval from 1 to 9 min helped us to identify compounds that are amino acids and sugars, as well as several phenolic compounds. Among the amino acids, the presence of L-lysine (**1**), L-tyrosine (**2**), proline (**3**), asparagine (**5**), and L-glutamic acid (**4**) was noted. From the class of sugars (retention time 1.65 min), α-lactose was identified (**6**). The remaining sugars were classified by us as unidentified compounds because the methodology used does not always allow for their accurate identification. In the retention time interval from 1 to 9 min, phenolic compounds such as melilotoside (**8**) and chlorogenic (**9**) and quinic (**12**) acids were also registered. Melilotoside (**10**) is *trans*-β-glucosyl-2-hydroxycinnamic acid, and for *S. hypericifolia* in particular and for the genus *Spiraea* in general, it was reported for the first time; however, in other plants of the Rosaceae family, melilotoside has been found previously [13].

Chlorogenic (**9**) and quinic (**12**) acids are some of the most widespread phenolic compounds in the plant kingdom and are known for their antioxidant properties [14]; in addition, chlorogenic acid is used by plants for the biosynthesis of lignin, which makes tissues resistant to any type of invasion [15]. Earlier, chlorogenic acid was detected in samples of *S. hypericifolia*, and the same is true for *p*-coumaric (**7**) and caffeic (**11**) acids [6,16,17]; data on quinic acid were presented for the first time.

This work was not aimed at exhaustive extraction and complete metabolomic analysis, but this LC-HRMS technique proved to be well suited for assaying flavonoids, of which 23 compounds were identified in the aqueous-ethanol extract.

**Figure 2:**
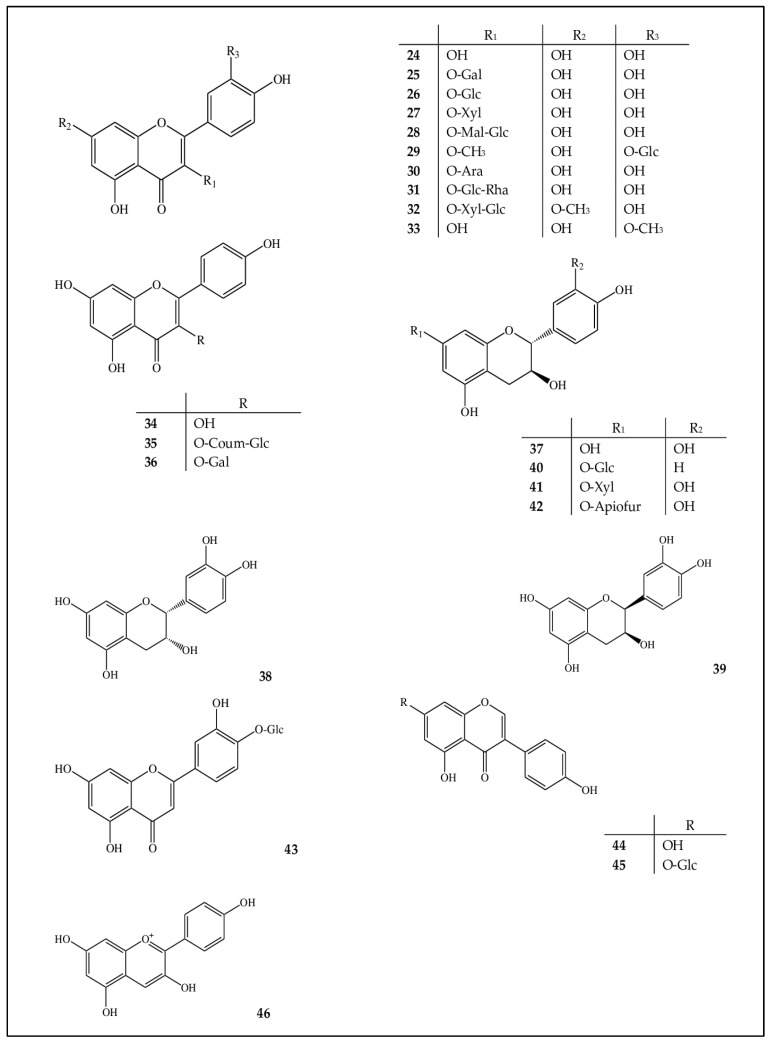
Structures of compounds **24**–**46** in *S. hypericifolia.* Ara: arabinopyranose, Gal: galactopyranose, Xyl: xylopyranose, Glc: glucopyranose, Rha: rhamnopyranose, Gal: galactopyranose, Mal-Glc: malonyl-glucopyranose, Apiofur: apiofuranose, and Coum-Glc: coumaroyl-glucopyranose.

Most of the identified flavonoid substances are flavonols, 10 of which are quercetin and its derivatives. Among these compounds, only isorhamnetin (**33**) and 3-*O*-methylquercetin-3′-*O*-β-D-glucopyranoside (**29**) are methyl-containing derivatives of quercetin (**26**). 3-*O*-methylquercetin-3′-*O*-β-D-glucopyranoside, just as most of the detected substances, is one of various quercetin glycosides. The carbohydrate moiety of these compounds is represented by mono- and disaccharides. Among the monosaccharides, pentoses (arabinose and xylose) and hexoses (galactose and glucose) were identified, and among the disaccharides, rutinose. The carbohydrate moiety is attached at the C-3 position (Figure 2). We reported on quercetin and its derivatives from *S. hypericifolia* in more detail earlier [12].

In another family of flavonols identified in the extract of *S. hypericifolia*—derivatives of kaempferol: tiliroside and trifolin—the carbohydrate residue was also attached at the C-3 position, whereas in the only detected flavone (luteolin-4′-*O*-β-D-glucopyranoside), it was attached at the C-4′ position.

The catechins found by us in *S. hypericifolia* were present in isomeric forms corresponding to catechin (**39**) and epicatechin (**38** and **39**). Additionally, glycosyl derivatives of catechins were identified (**40**–**42**), whose sugar moiety was represented by xylose, apiose, and glucose.

Genistein (**44**) and its glucoside genistin (**45**) belong to the family of isoflavones. It is believed that genistin and other glycosylated isoflavones are not biologically active but are activated only after being enzymatically separated into an aglycone and a carbohydrate residue; accordingly, their ratios in extracts are known to vary [18].

Pelargonidin (**46**) was also identified in the leaf extract of *S. hypericifolia*. This substance is among the six most common anthocyanidins, which are of great importance as antioxidants [19]. Its structure is one of the most stable because, in terms of substituents on the B ring, it has only one hydroxyl group (at the C-4′ position); the presence of additional methoxyl and hydroxyl groups considerably reduces the stability of the aglycone [20].

The presence of quercetin, hyperoside, isoquercitrin, rutin, and avicularin in leaf extracts of *S. hypericifolia* has previously been shown by many authors [6,7,8]. Aside from the aforementioned flavonols, researchers have identified in *S. hypericifolia* isomeric forms of epicatechin and catechin along with various forms of glycosides of the latter [21,22,23,24]. On the other hand, the flavones identified by T.K. Chumbalov et al. [24] in *S. hypericifolia*—apigenin, luteolin, apigenin 5-β-d-glucopyranoside, and luteolin-5-β-d-glucopyranoside—were not found by us. The only flavone that we identified here was luteolin-4′-*O*-β-d-glucopyranoside (**43**).

The presence of flavonoids has been noticed by other investigators in *S. hypericifolia* and in many other species of the genus *Spiraea* [25]. For instance, the following compounds have been found most often: quercetin and kaempferol (in 21 and 18 *Spiraea* species, respectively); hyperoside (in 14 species); avicularin, rutin, isoquercitrin, and isorhamnetin (in 9 species); catechin and epicatechin (in 4 species); and tiliroside and trifolin (in 2 species). (2S,3R)-3,5-Dihydroxy-2-(4-hydroxyphenyl)-3,4-dihydro-2H-chromen-7-yl-β-D-glucopyranoside (**40**) was detected in the genus *Spiraea* for the first time.

The family of fatty acids and their derivatives in the extract of *S. hypericifolia* is represented by compounds that are widespread in the plant kingdom. Monounsaturated palmitoleic acid (**14**), ω-3-unsaturated α-linolenic acid (**17**), and a member of the class of saturated carboxylic acids (azelaic acid) (**18**) were detected by us, as were amide derivatives of oleic and erucic acids: oleamide (**19**) and erucamide (**20**), respectively. Additionally, 12-oxo-phytodienoic acid (**16**) was identified, which is the initial precursor of jasmonic acid, which activates various defense responses and growth processes in tissues [26]. In the same category of the metabolite profile, a compound from the shikimate pathway was found here: gallic acid (**10**). The pentacyclic triterpenoids in this extract were represented by 18-β-glycyrrhetinic acid (**22**) (retention time 37.31 min) and 3-acetyl-11-keto-β-boswellic acid (**23**) (retention time 43.88 min). In studies by A. F. Raja et al., the antibacterial activity of 3-acetyl-11-keto-β-boswellic acid toward *Staphylococcus epidermidis* and *S. aureus* was confirmed [27], and neuroprotective properties of this triterpenoid were registered in vivo and in vitro [28].

### 2.2. HPLC-Based Quantification of Individual Phenolic Compounds in the Extract from S. hypericifolia

**Figure 3:**
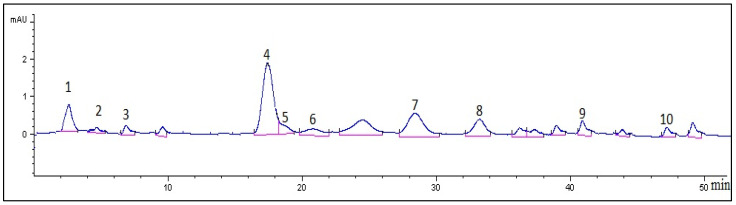
The chromatogram at 360 nm for the 70%-ethanol extract of the aboveground part of *S. hypericifolia*. On the horizontal axis: retention time in minutes; on the vertical axis: the detector’s signal (optical-density units). 1: Chlorogenic acid (t_R_ 3.2 min); 2: caffeic acid (t_R_ 4.4 min); 3: *p*-coumaric acid (t_R_ 7.8 min); 4: hyperoside (t_R_ 18.9 min); 5: isoquercitrin (t_R_ 19.8 min); 6: rutin (t_R_ 20.5 min); 7: avicularin (t_R_ 28.2 min); 8: astragalin (t_R_ 32.9 min); 9: quercetin (t_R_ 40.9 min); 10: kaempferol (t_R_ 47.8 min).

The quantitation of some identified phenolic compounds in the extract of *S. hypericifolia* by HPLC indicated that hyperoside (20.56 mg/[g of DE]) and avicularin (11.1 mg/[g of DE]) are among the major flavonoid compounds in this extract (Figure 3). Astragalin (5.38 mg/[g of DE]), rutin (3.41 mg/[g of DE]), chlorogenic acid (3.22 mg/[g of DE]), isoquercitrin (2.65 mg/[g of DE]), quercetin (2.26 mg/[g of DE]), *p*-coumaric acid (1.59 mg/[g of DE]), kaempferol (1.08 mg/[g of DE]), and caffeic acid (0.93 mg/[g of DE]) are also likely to make a substantial contribution to biological activities of the extract. Previously, by HPLC, in extracts from *S. hypericifolia* from other populations, we detected the same metabolites, except for gentisic acid, nicotiflorin, cinnamic acid, luteolin, and apigenin [6,12]. In the previous work, the main metabolites in the extract were also flavonoids: hyperoside (15.64 mg/[g of DE]), quercetin (6.62 mg/[g of DE]), isoquercitrin (6.56 mg/[g of DE]), and avicularin (9.76 mg/[g of DE]) [6,10], whose levels are comparable to the concentrations of metabolites in the extract under study.

### 2.3. Levels of Biologically Active Compounds in the S. hypericifolia Extract

**Figure 4:**
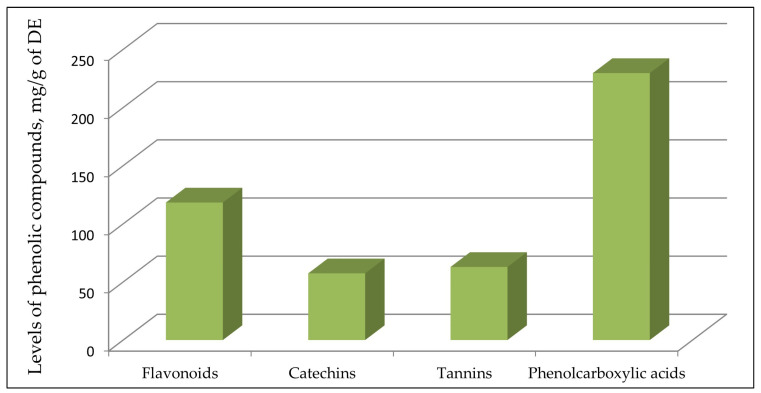
Levels of phenolic compounds in the extract of *S. hypericifolia*.

In addition to determining the profile and levels of metabolites, we measured concentrations of biologically active phenolic compounds. Phytochemical analysis of the aqueous-ethanol extract of *S. hypericifolia* revealed the predominance of phenolcarboxylic acids (229.6 mg/[g of DE]) and flavonoids (118.3 mg/[g of DE]) (Figure 4). The levels of tannins and catechins were comparable to them (62.9 mg/[g of DE] and 57.4 mg/[g of DE], respectively).

Our results on the concentrations of biologically active compounds in the extract from the aerial part of *S. hypericifolia* are partially consistent with the literature data. For instance, in separate analyses of the bark, stems, leaves, and root cores of *S. hypericifolia*, N.D. Storozhenko [8] found that the main biologically active compounds in the organs of the aboveground and underground parts of the plant are flavonoids: flavonols, flavans, and flavones. The highest total content of flavonoids was registered in the bark of the plant (44.1 mg/g): the concentration of flavones and their glycosides was 39.5 mg/g, and the concentration of catechin glycosides was 3.2 mg/g. The major biologically active compounds of the leaves of *S. hypericifolia* were flavonols and catechins, whose levels reached 32.6 and 1.8 mg/g, respectively [8]. By contrast, according to research by A.A. Kudaibergen et al. [3], the concentration of flavonoids in the underground part of *S. hypericifolia* is 53.6 mg/(g of total extract), while their concentration in the aboveground part does not exceed 7.7 mg/g. The discrepancy in the results is possibly due to the fact that the accumulation of these substances is influenced by environmental factors associated with the ontogenetic state of the plant at the time of sampling of the plant material; another possible reason is the choice of methods for analyzing the extracts.

### 2.4. Antioxidant Activity of the S. hypericifolia Extract

Previously, the high antiviral potential of the same kind of *S. hypericifolia* extract against human influenza virus and avian influenza virus was reported [9]. The antioxidant activity toward the DPPH radical (IC_50_ = 102.95 µg/[mL of DE]) of this extract turned out to be moderate. This plant’s antioxidant potential previously determined by us at other sampling sites proved to be slightly higher than that of the extract under study here. For example, in one paper, the half-maximal inhibitory concentration of an extract from *S. hypericifolia* against the DPPH radical was IC_50_ = 87.67 µg/mL [6]; in another study, DEs from the aerial part of *S. hypericifolia* showed higher antiradical activity (IC_50_ = 65.7 and 57.9 mg/mL) [10]. The antiradical activity of the powerful antioxidants trolox (IC_50_ = 7.74 μg/mL) and ascorbic acid (IC_50_ = 8.69 μg/mL) was found to be 11 and 13 times stronger than that of the analyzed extract. Our metabolomic analysis, which was performed on a plant from the genus *Spiraea* for the first time, showed the presence of highly pharmacologically active phenolic compounds in the extract from *S. hypericifolia*. This explains the observed effects of the extract under study.

The strong antioxidant properties of extracts of *Spiraea* plants have been confirmed several times and are usually correlated with high levels of polyphenols [29,30,31,32,33,34,35]. For instance, an 80% aqueous-ethanol extract from *S. canescens* D. Don., just as its individual ethyl acetate and butanol fractions, has shown strong biological effects: DPPH radical–scavenging activity (90.13%) and superoxide anion–scavenging activity (91.04%) [29]. There is also evidence of antioxidant activity in a methanol extract from *S. fritschiana* Schneid., which increases with the increasing concentration of the extract sample [30] and in aqueous and aqueous-ethanol extracts from *S. betulifolia* Pall., *S. beauverdiana* Schneid., *S. humulis* Pojark., *S. salicifolia* L., *S. pubescens* Turcz., *S. media* F. Schmidt, and *S. crenata* L. [10,32]. The seeds of *S. tomentosa* L. have a confirmed antioxidant activity too [36].

Among the biologically active compounds extracted from *Spiraea* species, there are substances possessing a strong antioxidant activity [37]. To date, the antioxidant properties of quercetin have been studied the most [38,39,40]. Many research articles are devoted to quercetin derivatives, such as rutin [41,42], isorhamnetin [43], quercitrin [44], isoquercitrin [45], hyperoside [46], reynoutrin [47], and others. A representative of isoflavones, genistein (found in *S. hypericifolia*), is also widely used as an antioxidant [48,49], as are catechins [50], whose antioxidant properties exceed those of, for example, vitamins E and C [51]. Due to the hydroxyl group in the gallate moiety, epigallotechins are more effective free-radical scavengers than other standard antioxidants, such as tocopherol and trolox [52,53].

Currently, the above-mentioned phenolic compounds are considered essential components of medicinal, cosmetic, pharmaceutical, and other products, because of the wide range of their biological activities [54]. Some examples include anti-inflammatory [55], antifungal [56], antimicrobial [57], antiviral [58], and other types of effects. The most intriguing are the reducing/oxidative properties of flavonoids since these substances are reported to be effective, have weak toxicity, and are widely applied in practice for the prophylaxis and therapy of diseases linked with oxidative stress [59,60,61]. Thus, the above findings about the profile of metabolites and antioxidant activity in *S. hypericifolia* indicate that these experiments are worthwhile and open up broad prospects for further comprehensive research on this plant.

## 3. Materials and Methods

### 3.1. Plant Material and Preparation of the Extract

Generative leafy shoots of *S. hypericifolia* were collected during the flowering period near village Ilyichevo, Shushenskiy District, Krasnoyarsk Krai (Siberia, Russia). The material was collected from 10–15 typical specimens of *S. hypericifolia*. Each analyzed sample contained 5 g of dry weight. The plant under study was collected and identified by an expert on the genus *Spiraea* in Russia: V.A. Kostikova, Ph.D., a senior researcher at the Central Siberian Botanical Garden SB RAS using the phenotype and morphological traits. Voucher specimens (No. SH-KI-25, No. SH-KI-26, and No. SH-KI-27) were deposited in the Plant Material Storage Room in the Laboratory of Phytochemistry, the Central Siberian Botanical Garden SB RAS (Novosibirsk, Russia). Whole annual branches of *S. hypericifolia* with leaves and flowers were used for the extraction of metabolites. Air-dried plant material was mechanically ground up to obtain a homogeneous powder with particle size down to 2–3 mm. The DE was prepared as follows: 5 g of aerial parts of plants was extracted three times with 70% ethanol at 60 °C for 8 h at a 1:20 herbal sample/solvent ratio for the first extraction and a 1:15 ratio for the second and third extractions. After cooling, the combined filtrates were concentrated in a rotary evaporator to remove the solvent, and then the thick extracts were dried in a vacuum drying cabinet to 5% residual moisture [12]. To determine concentrations of phenolic compounds, 10 g of the DE was dissolved in 10 mL of 70% ethanol.

### 3.2. LC-HRMS Analysis of Metabolites in the S. hypericifolia Extract

LC-HRMS was conducted at the Core Facility of Mass Spectrometric Analysis at the Institute of Chemical Biology and Fundamental Medicine SB RAS (Novosibirsk, Russia).

An Ultimate 3000 liquid chromatograph (Thermo Fisher Scientific, San Jose, CA, USA) coupled with a Q Exactive HF mass spectrometer (Thermo Fisher Scientific) was utilized to determine metabolomic profiles of the *S. hypericifolia* extract. The chromatographic separation was attained at a 0.4 mL/min flow rate on a Zorbax Eclipse XDB-C8 reversed-phase column (150 × 3.0 mm, 5 μm, Agilent Technologies, Santa Clara, CA, USA) thermostatted at 40 °C. The mobile phase was composed of 0.1% aqueous formic acid (eluent A) and acetonitrile (eluent B). The elution gradient was implemented as follows: from 5% to 70% B for 40 min, followed by an increase to 90% B for 8 min, a decrease to 5% B for 5 min, and re-equilibration under the initial conditions for 7 min [62].

The settings of the electrospray ionization (ESI) source were as follows: electrospray voltage: 3.2 kV in the negative mode and 4.2 kV in the positive mode; capillary temperature: 320 °C; and the S lens RF level: 50. Data were obtained by full-scan data-dependent acquisition (FS-dd-MS2) in the positive and negative modes at resolving power of 45,000 full-width at half maximum (FWHM) for *m*/*z* 200. The following settings of the mass spectrometer were employed: scan range: *m*/*z* 80–1200; automatic gain control (AGC): 3 × 10^6^; injection time: 100 ms; and the isolation window: *m*/*z* 2.0. The normalized collision energy for the fragmentation of molecular ions was set to 40 eV. A targeted tandem mass-spectrometry (MS/MS, i.e., dd-MS2) analysis was performed in both positive and negative modes at 15,000 FWHM (*m*/*z* 200). AGC for dd-MS2 was set to 1 × 10^5^, with injection time of 50 ms and a loop count of 5. In the section of dd settings, the AGC target was programmed at 8 × 10^3^, and maximum injection time was set to 100 ms. The data were analyzed using Xcalibur 4.0 and Compound Discoverer 3.1 software (Thermo Fisher Scientific). All the samples, including blank samples, were assayed in triplicate. All the samples were processed in Compound Discoverer 3.1 via a common workflow called “Environmental Unknown ID w Online and Local Database Searches.” Mass tolerance of 5 ppm was applied to all nodes. Several databases, i.e., KEGG (https://www.genome.jp/kegg/; accessed on 10 March 2021), MassBank (https://massbank.eu/MassBank/; accessed on 10 March 2021), PlantCyc (https://plantcyc.org/; accessed on 10 March 2021), and Planta Piloto de Quimica Fina Universidad de Alcala (http://www.cqab.eu/index.php/en/; accessed on 10 March 2021), were chosen in ChemSpider. A more detailed procedure for identifying substances is described in Ref. [62].

Metabolites were identified on the basis of both accurate mass and fragment mass “fingerprint” spectra via searches against the spectra of compounds available in the mzCloud database (https://www.mzcloud.org; accessed on 10 June 2023). If compounds were absent in mzCloud, they were tentatively identified using a ChemSpider search. According to the workflow, the masses extracted from the chromatograms were aligned and filtered to remove (i) background compounds present in the blank sample, (ii) substances that failed to become fragmented, (iii) compounds’ masses that were absent in the databases, and (iv) signals with low intensity.

### 3.3. Quantification of Individual Phenolic Compounds by HPLC

Analysis of levels of phenolic compounds was performed by means of an Agilent 1200 HPLC system equipped with a diode array detector and a system for the collection and processing of chromatographic data: ChemStation (Agilent Technologies). The separation was performed on a column Zorbax SB-C18 (5 µm, 4.6 × 150 mm) at 26 °C. The methanol content of the mobile phase in an aqueous solution of phosphoric acid (0.1%) was varied from 33% to 100% during 53 min. The eluent flow rate was 1 mL/min. Detection was executed at wavelengths λ = 255, 270, 340, and 360 nm. Quantitative analyses of flavonoids were performed by an external standard method [63].

### 3.4. Quantification of Biologically Active Metabolites in the Plant Extract 

The total flavonol content was determined spectrophotometrically by the AlCl_3_ method [64]. Briefly, 0.5 mL of 2% aluminum chloride (AlCl_3_) in ethanol was mixed with the same volume of plant extract. After 1 h, absorption readings at 415 nm were taken against a blank (ethanol). Optical density of the mixture was measured on an SF-56 spectrophotometer (Lomo, St. Petersburg, Russia). The concentration of flavonoids was found by means of calibration curves constructed with rutin as a standard. The results were expressed in milligrams of a rutin equivalent per gram of a DE.

The level of catechins was determined spectrophotometrically by a method based on the ability of catechins to produce a crimson coloration with a solution of vanillin in concentrated hydrochloric acid [65,66]. Optical density of the two solutions (extracted and standard solutions) was measured at 504 nm. The conversion factor was calculated using (±)-catechin. 

The concentration of hydrolyzable tannins was determined by a method of L.M. Fedoseeva [67]. An extract (10 mL) was transferred into a 100 mL volumetric flask, and 10 mL of a 2% aqueous solution of ammonium molybdate was introduced. The flask contents were brought to the nominal volume with purified water and incubated for 15 min. The intensity of the resulting color was measured on the SF-56 spectrophotometer (Lomo, Russia) at 420 nm in a cuvette with a light path of 1 cm. A government standard sample of tannin was employed for building a standard curve. The results were expressed in milligrams of tannin equivalent per gram of dry extract.

Total phenolic (hydroxycinnamic) acids were quantified according to J. Katanić et al. [68]. In brief, 5 mL of water was added to 1 mL of extract. Then, to the mixture, 1 mL of HCl (0.1 M), 1 mL of the Arnow reagent (10% [*w*/*v*] of sodium molybdate and 10% [*w*/*v*] of sodium nitrite), and 1 mL of NaOH (1 M) were added, and the volume was brought to 10 mL with water. Absorbance was read immediately at 490 nm. The conversion factor was calculated by means of caffeic acid.

### 3.5. Estimation of Antiradical Activity in a 1,1-Diphenyl-2-picrylhydrazyl (DPPH) Assay

Free-radical scavenging capacity of the samples was determined by the DPPH method [69,70] with modifications. For this purpose, a 2 mL aliquot of an extract (dissolved in 70% ethanol to concentrations in the range of 15–500 µg/mL) was mixed with 3 mL of a DPPH solution (62 mg/mL in ethanol). After 30 min of incubation in darkness at room temperature, optical density (A) was measured at 517 nm against a blank sample. Free-radical scavenging activity was calculated as percentage inhibition via the following formula:

I% = (A_blank_ − A_sample_/A_blank_) × 100 (1)

where A_blank_ is optical density of a control solution (containing all reagents except the tested extract), and A_sample_ is optical density of the sample.

The results were expressed in IC_50_ defined as the concentration of an antioxidant that causes 50% DPPH loss in the DPPH radical scavenging activity assay. 6-Hydroxy-2,5,7,8-tetramethylchroman-2-carboxylic acid (trolox) and ascorbic acid (AA) solutions (2.5–50.0 mg/mL) served as positive controls.

### 3.6. Chemicals

All chemicals were of MS or analytical grade. Chemical reference standards of chlorogenic acid, caffeic acid, quercetin, and isoquercitrin were purchased from Sigma-Aldrich (Taufkirchen, Germany), whereas rutin, avicularin, and hyperoside were from Fluka Chemie AG (Buchs, Switzerland), and *p*-coumaric acid was from Serva (Heidelberg, Germany).

### 3.7. Statistical Analysis

This analysis was carried out in GraphPad Prism v8.4.3 and Microsoft Excel 2016. All samples, including blank samples, which consisted of the pure solvent, were analyzed as two biological replicates with three technical replicates per treatment group.

## 4. Conclusions

In the aqueous-ethanol extract from *S. hypericifolia*, we identified 47 biologically active compounds, including 23 flavonoids by LC-HRMS. An HPLC analysis revealed that hyperoside and avicularin are among the major flavonoid compounds in the extract. Astragalin, rutin, isoquercitrin, quercetin, and kaempferol are also likely to make a substantial contribution to the biological activity of the extract. The aqueous-ethanol extract from *S. hypericifolia* possesses antioxidant activity, which is probably attributable to high concentrations of phenolcarboxylic acids, flavonoids, and tannins.

## Figures and Tables

**Table 1:** Metabolites in the aqueous-ethanol extract from *S. hypericifolia* according to LC-HRMS with databases mzCloud and ChemSpider.

ID	Identified Compounds	t_R_ (Min)	Mode	Calculated Mass	Measured Mass	Delta Mass (Da)	Delta Mass (ppm)	MzCloud Score
*Amino acids*								
1	L-Lysine (LYS)	1.49	positive	146.10568	146.10553	0.00015	1.04	85.1
2	L-Tyrosine (TYR)	1.78	positive	181.07388	181.07389	−0.00002	−0.09	96.5
3	Proline	1.68	positive	115.06355	115.06333	0.00022	1.89	99.8
4	L-Glutamic acid	1.56	negative	147.05281	147.05316	−0.00035	−2.36	80.7
5	Asparagine	1.60	positive	132.05366	132.05349	0.00016	1.24	93.5
*Sugars*								
6	α-Lactose	1.65	positive	342.11602	342.11621	−0.00020	−0.57	76.8
*Phenylpropanoids*								
7	*p*-Coumaric acid *	11.88	positive	164.04747	164.04734	0.00013	0.78	99.5
8	Melilotoside	1.91	positive	326.10042	326.10017	0.00025	0.76	98.7
9	Chlorogenic acid *	8.79	negative	354.09479	354.09508	−0.00029	−0.83	-
10	Gallic acid	51.47	negative	170.02207	170.02152	0.00055	3.23	-
11	Caffeic acid *	9.50	negative	180.04149	180.04226	−0.00077	−4.27	90.9
12	(−)-Quinic acid	1.70	positive	192.06355	192.06339	0.00016	0.82	75.3
*Aromatic polyketides*								
13	Shogaol	28.35	positive	276.17540	276.17254	0.00285	10.34	89.5
*Fatty acids and derivatives*								
14	Palmitoleic Acid	45.38	positive	254.22452	254.22458	−0.00006	−0.24	86.7
15	9-Oxo-10,12-octadecadienoic acid	39.24	positive	294.21936	294.21949	−0.00014	−0.46	97.7
16	12-Oxo-phytodienoic Acid	36.86	positive	292.20379	292.20384	−0.00005	−0.19	90.8
17	α-Linolenic acid	45.52	positive	278.22446	278.22458	−0.00012	−0.45	98.6
18	Azelaic acid	16.25	negative	188.10423	188.10486	−0.00063	−3.35	97.5
19	Oleamide	47.07	positive	281.27176	281.27186	−0.00011	−0.38	98.9
20	Erucamide	44.72	positive	337.33445	337.33447	−0.00002	−0.05	96.4
*Fatty alcohols*								
21	Mannitol	1.59	negative	182.07820	182.07904	−0.00083	−4.58	90.5
*Triterpenoids*								
22	18-β-Glycyrrhetinic acid	37.31	positive	470.33975	470.33961	0.00014	0.30	82.3
23	3-Acetyl-11-keto-β-boswellic acid	43.88	positive	512.35030	512.35017	0.00013	0.25	61.9
*Flavonols*								
24	Quercetin *	12.40	positive	302.04243	302.04265	−0.00022	−0.73	99.9
25	Hyperoside *	12.39	positive	464.09520	464.09548	−0.00027	−0.59	99.6
	(quercetin-3-galactoside)							
26	Isoquercitrin *	12.61	negative	464.09548	464.09548	0.00000	0.00	99.4
	(quercetin-3-*O*-β-d- glucopyranoside)							
27	Reynoutrin	13.72	positive	434.08471	434.08491	−0.00020	−0.45	98.4
	(quercetin-3-*O*-β-d- xylopyranoside)							
28	Quercetin-3-*O*-(6″-*O*-malonyl) -β-d-glucoside)	13.58	positive	550.09574	550.09587	−0.00013	−0.24	97.5
29	3-*O*-Methylquercetin-3′-*O*-β-d- glucopyranoside	14.10	positive	478.11095	478.11113	−0.00018	−0.37	97.6
30	Avicularin * (quercetin-3-*O*-α-l- arabinopyranoside)	14.25	negative	434.08417	434.08491	−0.00074	−1.71	-
31	Rutin *	9.22	negative	610.15368	610.15338	0.00029	0.48	-
32	Rhamnetin-3-*O*-β-d-xylopyranosyl- β-d-glucopyranoside	12.48	positive	610.15339	610.15338	0.00001	0.01	96.9
33	Isorhamnetin	14.10	positive	316.05826	316.05830	−0.00004	−0.13	99.0
	(3′-methylquercetin)							
34	Kaempferol	13.55	positive	286.04768	286.04774	−0.00006	−0.21	99.5
35	Tiliroside (kaempferol-3(*p*-coumaroyl) glucoside)	18.50	positive	594.13731	594.13734	−0.00003	−0.06	98.9
36	Trifolin	13.55	positive	448.10041	448.10056	−0.00015	−0.34	99.8
	(kaempferol-3-*O*-β-d- galactoside)							
*Flavans*								
37	Catechin *	2.19/6.57/8.11	positive	290.07922	290.07904	0.00018	0.62	99.1
38	(−)-Epicatechin	2.19/6.57/8.11	positive	290.07924	290.07904	0.00021	0.71	99.0
39	(+)-Epicatechin	2.19/6.57/8.11	positive	290.07924	290.07904	0.00021	0.71	98.8
40	(2S,3R)-3,5-Dihydroxy-2-(4- hydroxyphenyl)- 3,4-dihydro-2*H*-chromen-7-yl β-d-glucopyranoside	8.11	positive	436.13687	436.13695	−0.00008	−0.19	64.2
41	(+)-Catechin-7-*O*-β-d-xyloside	1.80	positive	422.12125	422.12130	−0.00005	−0.11	78.2
42	Catechin 7-*O*-apiofuranoside	1.92	positive	422.12111	422.12130	−0.00019	−0.44	76.2
*Flavones*								
43	Luteolin- 4′-*O*-β-d-glucopyranoside	11.36	positive	448.10046	448.10056	−0.00010	−0.22	98.8
*Isoflavones*								
44	Genistein	1.88	positive	270.05287	270.05822	0.00005	0.19	99.8
	(5,7,4′-trihydroxyisoflavone)							
45	Genistin	13.08	positive	432.10574	432.10565	0.00010	0.22	98.6
	(genistein-7-*O*-β-d- glucopyranoside)							
*Anthocyanidins*								
46	Pelargonidin	13.09	positive	270.05276	270.05282	−0.00006	−0.22	99.8
*Chalcones*								
47	Benzophenone	31.10	positive	182.07306	182.07316	−0.00010	−0.058	87.6

Note: * compounds confirmed by means of standards; “-”: ChemSpider only. Compounds **28** and **29** have also been detected by us before [6,12].

## Data Availability

Raw data are available upon request.
